# Perioperative Costs of Elective Surgical Procedures in Medicare Advantage Compared With Traditional Medicare

**DOI:** 10.1001/jamahealthforum.2025.2258

**Published:** 2025-08-01

**Authors:** Eran Politzer, Timothy S. Anderson, John Z. Ayanian, Vilsa E. Curto, Jeffrey Souza, Thomas C. Tsai, Bruce E. Landon

**Affiliations:** 1 Department of Health Care Policy, Harvard Medical School, Boston, Massachusetts; 2 Federmann School of Public Policy and Governance, The Hebrew University of Jerusalem, Israel; 3 Division of General Internal Medicine, University of Pittsburgh, Pittsburgh, Pennsylvania; 4 Institute for Healthcare Policy and Innovation, University of Michigan, Ann Arbor; 5 Division of General Medicine, University of Michigan, Ann Arbor; 6 Department of Health Policy and Management, Harvard T.H. Chan School of Public Health, Boston, Massachusetts; 7 Department of Surgery, Brigham and Women’s Hospital, Boston, Massachusetts; 8 Division of General Medicine, Beth Israel Deaconess Medical Center, Boston, Massachusetts

## Abstract

**Importance:**

The utilization of elective surgical procedures is lower for patients enrolled in Medicare Advantage (MA) than in traditional Medicare (TM), but it remains unclear whether surgical episode costs differ between MA and TM for comparable patients.

**Objective:**

To compare the estimated costs, markers of resource use (eg, length of stay and location of surgery), and outcomes of surgical episodes for similar patients enrolled in MA and TM.

**Design, Setting, and Participants:**

In this retrospective cohort study, 2019 Medicare claims and encounters data were analyzed to compare differences in surgical episode costs for beneficiaries with MA vs TM who underwent common elective surgical procedures across 11 categories. Procedures performed in either inpatient or outpatient settings were included in the analysis. Data were analyzed from January 2023 to March 2025.

**Exposures:**

Enrollment in MA or TM.

**Main Outcomes and Measures:**

The primary outcomes were estimated 30-day costs of surgical episodes and factors affecting costs and/or outcomes, including share of inpatient procedures, length of stay, share of patients discharged home, and 30-day readmission rates. A secondary outcome explored potential facility selection and patient steering by estimating the distance traveled to surgery. Multivariable linear regression models adjusted for the type of surgical procedure, patient characteristics, and their Elixhauser Comorbidity Index were used to compare outcomes of surgical episodes in patients with MA vs TM within hospital referral regions.

**Results:**

The analysis included 1 177 700 surgical procedures among 1 110 263 Medicare beneficiaries (mean [SD] age, 73.42 [5.8] years; 686 708 females [58.3%]). The overall rate of surgery utilization was lower among MA patients vs TM patients (difference in rate, −4.4%; 95% CI, −4.8% to −4.1%), with variation found across surgical categories. Across procedures, 30-day surgical episode costs for MA patients vs TM patients were, on average, $671 (95% CI, $639-$702) lower. The share of procedures billed at the higher inpatient rate was 5.41 (95% CI, 5.23-5.58) percentage points (pp) lower for MA patients than for TM patients, and the mean length of inpatient stay was 0.27 (95% CI, 0.26-0.29) days shorter. The share of patients discharged home was higher for those with MA vs TM (3.82 [95% CI, 3.65-3.99] pp). MA patients traveled a mean of 2.32 (95% CI, 1.62-3.01) miles farther for surgery. Readmission rates were lower for patients with MA (−0.70 [95% CI, −0.83 to −0.58] pp).

**Conclusions and Relevance:**

This study found that in addition to lower utilization of common elective surgical procedures, the costs of surgical episodes were lower for patients enrolled in MA than those enrolled in TM. MA plans had lower costs because more procedures were performed in outpatient settings, required shorter lengths of stay, and less expensive postacute care, with no apparent harm to overall quality. Physician and surgical facility selection and patient steering likely contributed to these cost differences. These findings highlight potential mechanisms by which MA plans may achieve cost savings compared with TM plans.

## Key Points

**Question:**

Are the costs of elective surgical episodes lower in Medicare Advantage (MA) compared with traditional Medicare (TM) for similar patients?

**Findings:**

In this retrospective cohort study of 1.18 million surgical procedures among 1.11 million Medicare beneficiaries, estimated 30-day costs of surgery episodes were 3.1% lower for MA patients than for comparable TM patients. MA patients more often had outpatient surgeries, shorter inpatient hospital stays, received less postacute care, and had fewer readmissions.

**Meaning:**

The findings suggest that lower perioperative costs and resource use offer other potential mechanisms for cost savings in Medicare Advantage.

## Teaser

This cohort study compared estimated 30-day costs of surgical episodes for adults with Medicare Advantage vs traditional Medicare.

## Introduction

Spending on surgical episodes of care constitutes a substantial portion of Medicare’s total expenditure, accounting for 51% of traditional Medicare (TM) spending in 2014, totaling $125 billion.^1^ Previous studies have identified substantial variation in the costs of TM surgical episodes beyond differences due to geography or illness severity.^2^ This variation underscores the potential for cost savings through bundled payments,^3^ the selection of more cost-effective clinicians and facilities and the implementation of care and utilization management programs.

Prior research indicates that beneficiaries with Medicare Advantage (MA) have lower utilization rates of elective surgical procedures.^4,5^ However, evidence regarding the costs of surgical episodes is inconclusive. A persistent challenge in these studies is adjusting resource use to account for patient acuity. Moreover, several prior studies^4,5^ relied on data from over a decade ago, predating recent increases in MA enrollment, which now includes over 50% of eligible Medicare beneficiaries.^6^ Additionally, these studies often relied on limited samples.

We used individual-level Medicare inpatient, outpatient, postacute care, and professional claims and encounters data to examine the costs and outcomes of patients with MA undergoing elective surgical procedures compared with those enrolled in TM. We focused on common surgical procedures feasible in both outpatient and inpatient settings, potentially providing MA plans greater flexibility to use cost-reducing tools. The data also enabled us to adjust for differences between patients enrolled in MA or TM who underwent specific surgical procedures, facilitating a more precise comparison of perioperative resource utilization between MA and TM.

## Methods

We used TM Medicare claims data to identify a set of *Current Procedural Terminology* (*CPT*) codes for outpatient procedures and *International Statistical Classification of Diseases and Related Health Problems, Tenth Revision* (*ICD-10*) procedure codes for inpatient procedures for common nonminor elective surgical procedures feasible in both inpatient and outpatient settings. Subsequently, we identified all surgical procedures matching these codes in the 2019 MA encounters and TM claims files. Using this analytic dataset, we examined surgical procedures of comparable MA and TM patients who underwent surgical procedures within the same category, estimating the association of MA enrollment with the costs and outcomes of the surgical episodes. This cohort study was approved by the Centers for Medicare & Medicaid privacy board and the Harvard Medical School institutional review committee, which waived the informed consent requirement because the study used deidentified data. The Strengthening the Reporting of Observational Studies in Epidemiology (STROBE) reporting guideline was followed.

### List of Examined Surgical Procedures

Our analysis focused on a selection of common elective surgical procedures identified by *CPT* codes in 2019 claims data from the TM Carrier file for a 20% sample of TM beneficiaries. Each elective surgical procedure was classified into a surgical category using the corresponding clinical classification software (CCS) code, a classification system that groups *CPT* codes into categories provided by the Healthcare Cost and Utilization Project.^7^ We identified a set of common surgical procedures (with at least 20 000 procedures performed in their CCS category), avoiding overly minor surgical procedures (requiring a mean charge of at least $400 for each *CPT* code) and ensuring they were performed in both outpatient and inpatient settings (at least 10% in each setting). Our empirical approach to selecting the list of examined surgical procedures aligned with prior research relying on expert opinion.^8^

To identify procedures in facility claims, we determined the most relevant *ICD-10* procedure codes for each *CPT* code on our list. We linked 2019 professional claims from the TM Carrier file with their corresponding facility claims in the TM Fee-For-Service Inpatient Claim File and added *ICD-10* procedure codes to our list if they appeared at least 10 times in these linked claims, with at least 1 instance as the sole *ICD-10* procedure code on the inpatient claim.

To ensure that related surgical procedures were not omitted, we identified the most common diagnoses for patients undergoing each surgical procedure on our list. Then, using 2019 professional and facility claims, we identified additional related procedures that patients with these diagnoses underwent. These procedures might have represented alternative approaches that could serve as substitutes for the primary procedure identified. The related *CPT* codes and *ICD-10* procedure codes were then added to our main list of surgery codes. Lastly, we adjusted the categories of surgical procedures when the CCS classification was excessively broad or narrow.

### Analytic Sample of MA and TM Procedures

The analytic sample included all elective procedures that matched our list of surgery codes for patients aged 65 years or older. These procedures were identified either in the inpatient or outpatient facility files of TM claims or MA encounters, using a 100% sample of Medicare beneficiaries. Additionally, procedures performed in stand-alone ambulatory surgery centers (ASCs) were included if they appeared in the MA or TM Carrier files for a 20% sample of beneficiaries. To account for the difference in sample sizes, ASC procedures were assigned a sample weight 5 times higher than other procedures.

### Patient Characteristics

We obtained patient demographic information from the 2019 Master Beneficiary Summary File, including age, sex, race and ethnicity determined by the Research Triangle Institute algorithm, zip code of residence, Medicaid enrollment status, and the reason for original Medicare eligibility (age, disability, or end-stage kidney disease). Race and ethnicity data were included in the analysis because of the racial and ethnic disparities in health that may affect surgery outcomes. Racial and ethnic categories included Black, Hispanic, White, and other (not defined) race and ethnicity. Additionally, we identified the hospital referral region (HRR) corresponding to the patient’s zip code. Based on *ICD-10* diagnosis codes from the index surgery claim or encounter, we calculated the Elixhauser Comorbidity Index for the risk of in-hospital mortality and Elixhauser Comorbidity Index for the risk of 30-day all-cause readmissions using Healthcare Cost and Utilization Project software, v2024.1.^9,10^ For the sensitivity analysis, we calculated the Centers for Medicare & Medicaid Services’ Hierarchical Condition Category (CMS-HCC) prospective risk score for each patient from the 20% sample, using 2018 diagnoses, excluding diagnoses derived from medical record reviews.^11,12^

### Outcomes of Interest

We identified a set of measures associated with the total costs of surgical episodes in MA plans. For each procedure, we determined the type of billing (inpatient or outpatient), the length of stay, whether the patient was discharged on the same day of the admission or the next day, and whether patients were discharged to their homes without additional postacute care. Based on the surgical procedure code (*CPT* codes for outpatient procedures and *ICD-10* codes for inpatient procedures), we also distinguished whether the procedure used an open approach or a minimally invasive approach. Additionally, we tracked instances of patient readmissions, defined here as a visit to an emergency department or nonelective inpatient care within 30 days of their discharge, as well as 30-day mortality rates. To explore whether patient steering to specific surgical facilities may serve as a mechanism for potential cost savings, we calculated, for each surgical procedure, the geographical distance between the centroids of the surgical facility’s zip code and the patient’s zip code.

### Estimation of 30-Day Costs

We estimated the 30-day costs of surgical episodes by first summing costs for the 20% sample of TM beneficiaries for whom complete utilization and cost data were available. Costs included professional, facility, and postacute care charges paid by Medicare, the beneficiary, or any other insurer within 30 days from the episode’s start, separating costs incurred during the surgical stay and those incurred afterward. A summary of these costs by surgery group is provided in eTable 1 in Supplement 1.

Next, we estimated 2 linear regression models for each surgical category, one for costs during the surgical stay and another for costs after the stay. All models controlled for specific surgical codes, an interaction term for inpatient billing and the length of stay, whether the patient was discharged home, and whether the patient was readmitted, as well as patient age, sex, race and ethnicity, HRR, Elixhauser Comorbidity Index for the risk of in-hospital mortality, and Elixhauser Comorbidity Index for the risk of 30-day all-cause readmissions, indicators for the original reason for Medicare entitlement, and whether the patient had dual eligibility for Medicare and Medicaid. Models for costs during the stay were additionally controlled for inpatient death. For costs after discharge, models controlled for patient death within 30 days of admission. These costs were set to zero for patients who died during the surgical stay.

Using the models’ estimated coefficients and the characteristics of each surgical episode, we then estimated 30-day costs for all procedures in the analytic sample. This included surgical procedures for TM patients who were excluded from the 20% sample (professional claims were unavailable) and all surgical procedures for MA patients (when cost data were unavailable).

### Statistical Analysis 

We used multivariable linear regression models to estimate the weighted correlations between MA enrollment and various outcomes, including distance traveled to the surgical facility, the percentage of inpatient procedures, length of stay (for the entire sample and separately for inpatient procedures), the proportion of discharges within 1 calendar day, the proportion of patients discharged home (for the entire sample and separately for inpatient or outpatient procedures), the 30-day mortality rate, the percentage of readmitted patients within 30 days, and the estimated 30-day costs. For surgical categories with an available minimally invasive alternative, we estimated the correlation of MA enrollment with the proportion of procedures conducted using an open approach. We repeated all these analyses also for each surgical category separately.

Our baseline analysis included controls for the surgical category, patient age, sex, race, whether a patient had dual Medicare-Medicaid eligibility, the original reason for Medicare entitlement, and the patient’s HRR. Additionally, we controlled for the Elixhauser Comorbidity Index for mortality except when examining readmissions, which was controlled for using the Elixhauser Comorbidity Index for 30-day all-cause readmissions. For the 30-day cost analysis, we controlled for Elixhauser Comorbidity Index for the risk of in-hospital mortality and the Elixhauser Comorbidity Index for the risk of 30-day all-cause readmissions. Analyzing the distance patients traveled to undergo their surgical procedure, we controlled for patients’ zip codes instead of their HRR. As a sensitivity analysis, we estimated a specification controlling for surgical codes instead of categories.

Recognizing that differences in our examined outcomes, which include costs and factors that affect costs between MA and TM procedures, could arise both from the selection of hospitals and facilities in MA networks and from different outcomes for MA patients within the same facility, we estimated a specification that included a fixed effect for the facility where the procedure was performed. In addition, we conducted 2 sensitivity analyses to explore possible unmeasured differential selection for surgical procedures. The first analysis examined a different sample of nonelective urgent procedures from our list of surgical procedures and the second focused on a subgroup of patients with dual Medicare-Medicaid eligibility. Lastly, we calculated age- and sex-standardized rates of surgery utilization in MA patients and TM patients for each surgical category. Data were analyzed from January 2023 to March 2025 using SAS, version 9.4 (SAS Institute Inc) or Stata, version 18.0 (StataCorp LLC).

## Results

A total of 1 177 700 procedures were performed on 1 110 263 Medicare beneficiaries (686 708 females [58.3%] and 491 002 males [41.7%]; mean [SD] age, 73.4 [5.8] years). Our list of surgical procedures comprised 11 surgical categories. The list included 64 *CPT* codes and 177 *ICD-10* procedure codes. The analytic sample represented all the procedures with these codes conducted in 2019, with 425 576 procedures for MA patients and 752 124 for TM patients. Surgical patients enrolled in MA or TM in our sample were similar in age, but the proportion of female patients enrolled in MA was higher (2.5% [95% CI, 2.3%-2.7%] percentage points [pp]), the share of White patients lower (−9.9 [95% CI, −10.1 to −9.8] pp), the proportion of patients whose original eligibility for Medicare was due to disability was higher (4.3 [95% CI, 4.2-4.5] pp), and the share of patients with dual Medicare-Medicaid eligibility was higher (5.6 [95% CI, 5.5-5.7] pp) (Table 1).

**Table 1.** Characteristics of Surgical Patients by Medicare Program

Characteristic	All surgical procedures^a^	MA procedures	TM procedures	Absolute difference (95% CI)	Difference, %
Age, mean (SD), y	73.4 (5.8)	73.5 (5.8)	73.4 (5.7)	0.1 (0.1 to 0.1)	0.1
Sex, %					
Female	58.3	59.9	57.4	2.5 (2.3 to 2.6)	4.3
Male	41.7	40.1	42.6	−2.5 (−2.6 to −2.3)	−5.8
Race and ethnicity, %^b^					
Black	6.5	9.1	5.0	4.1 (4.0 to 4.2)	82.4
Hispanic	5.9	9.8	3.7	6.1 (6.0 to 6.2)	164.1
White	83.1	76.8	86.7	−9.9 (−10.1 to −9.8)	−11.4
Other	4.5	4.3	4.6	−0.3 (−0.4 to −0.2)	−5.9
Medicare eligible due to disability, %^c^	12.8	15.5	11.2	4.3 (4.2 to 4.5)	38.8
Dual Medicare-Medicaid eligibility, %	9.6	13.2	7.6	5.6 (5.5 to 5.7)	73.7
Elixhauser Comorbidity Index (mortality), mean (SD)^d^	−2.0 (5.7)	−2.1 (5.7)	−2.0 (5.7)	−0.1 (−0.1 to −0.0)	−2.3
Elixhauser Comorbidity Index (readmissions), mean (SD)^e^	1.4 (3.0)	1.4 (3.1)	1.4 (3.0)	0.1 (0.1 to 0.1)	6.0
Surgical episodes, No.^f^	1 177 700	425 576	752 124	NA	NA

Abbreviations: MA, Medicare Advantage; NA, not applicable; TM, traditional Medicare.

^a^ Procedures performed in 1 110 263 Medicare beneficiaries.

^b^ Race was ascertained using the Research Triangle Institute algorithm.

^c^ Share of patients whose original eligibility for Medicare was due to disability.

^d^ Elixhauser Comorbidity Index of in-hospital mortality. The score ranges from −19 to 89, with higher scores indicating a greater risk of in-hospital mortality.

^e^ Elixhauser Comorbidity Index of 30-day all-cause readmissions. The score ranges from −19 to 89, with higher scores indicating a greater risk of readmission.

^f^ Patients may have multiple surgical episodes if they have more than one of the included surgical procedures during 2019.

### Surgery Rates

Age- and sex-standardized rates of surgery were lower in MA beneficiaries than TM beneficiaries (difference in rate, −4.4% [95% CI, −4.8% to −4.1%]; 0.91 fewer surgical procedures per 1000 beneficiaries), with notable variations across surgical categories (Table 2). Surgery rates for MA patients were lower for the 3 most common categories: knee arthroplasties (difference in rate, −5.2% [95% CI, −5.8% to −4.6%]), spinal operations (difference in rate, −13% [95% CI, −13.8% to −12.4%]), and arm or shoulder arthroplasties (difference in rate, −14.3% [95% CI, −15.6% to −13.3%]), which together accounted for 74% of the sample. Conversely, surgery rates for patients with MA were higher for most other categories, including thyroidectomy (difference in rate, 6.8% [95% CI, 5.1% to 8.9%]) and prostatectomy (difference in rate, 14% [95% CI, 12.0% to 16.0%]). A weighted average of surgery rates by category that used surgery costs as weights revealed that lower rates of surgical procedures in MA patients led to an overall cost savings of 6.3% on the included surgical procedures compared with surgical procedures in TM patients.

**Table 2.** Age- and Sex-Standardized Surgery Rates, by Surgical Category and Medicare Program

Category	Total procedures, No.^a^	Rate, per 1000 MA beneficiaries^b^	Rate, per 1000 TM beneficiaries^b^	Absolute difference in rate per 1000 MA vs TM patients (95% CI)	Difference in rates, % (95% CI)
Total sample	1 177 700	19.52	20.42	−0.91 (−0.98 to −0.83)	−4.4 (−4.8 to −4.1)
Knee arthroplasty	486 823	8.04	8.48	−0.44 (−0.49 to −0.39)	−5.2 (−5.8 to −4.6)
Spinal operations	287 647	4.49	5.16	−0.67 (−0.71 to −0.64)	−13.0 (−13.8 to −12.4)
Arm or shoulder arthroplasty	97 910	1.54	1.80	−0.26 (−0.28 to −0.24)	−14.3 (−15.6 to −13.3)
Ventral or incisional hernia	83 604	1.54	1.41	0.13 (0.11 to 0.15)	9.2 (7.8 to 10.6)
Thyroidectomy	46 462	0.84	0.79	0.05 (0.04 to 0.07)	6.8 (5.1 to 8.9)
Prostatectomy	43 688	0.86	0.75	0.11 (0.09 to 0.12)	14.0 (12.0 to 16.0)
Mastectomy	36 512	0.69	0.62	0.07 (0.06 to 0.09)	11.4 (9.7 to 14.5)
Paraesophageal hernia	28 183	0.48	0.52	−0.04 (−0.05 to −0.02)	−7.1 (−9.6 to −3.9)
Hysterectomy	26 338	0.51	0.45	0.06 (0.05 to 0.07)	13.1 (11.1 to 15.6)
Nephrectomy	26 660	0.49	0.47	0.03 (0.01 to 0.04)	5.3 (2.1 to 8.5)
Liver procedures	13 873	0.25	0.25	0.01 (0.0 to 0.02)	3.7 (0.0 to 7.4)

Abbreviations: MA, Medicare Advantage; TM, traditional Medicare.

^a^ Weighted number of surgical procedures based on complete information on procedures in hospitals’ inpatient and outpatient settings for 100% of Medicare beneficiaries and information on procedures in stand-alone ambulatory surgery centers for a random sample of 20% of Medicare beneficiaries.

^b^ Surgery rates are adjusted for patients’ sex and 5-year age groups.

### Outcomes of Interest 

Multivariable linear regression models found that MA enrollment was correlated with a longer distance traveled to the surgical facility compared with similar TM patients residing within the same zip code and undergoing a surgical procedure from the same category (weighted correlation coefficient, 2.32 [95% CI, 1.62-3.01 miles]) (Table 3). The mean (SD) distance traveled by TM patients was 49.1 (185.1) miles.

**Table 3.** Correlation of Medicare Advantage (MA) Enrollment With Surgical Characteristics and Outcomes

Characteristic and outcome	TM^a^	Correlation coefficient of MA enrollment (95% CI)^b^	Magnitude of difference for MA correlation coefficient, % (95% CI)
Preadmission			
Distance to facility, mean (SD) miles^c^	49.1 (185.1)	2.32 (1.62 to 3.01)	4.7 (3.3 to 6.1)
During admission			
Share billed as an inpatient procedure, %	63.2	−5.41 (−5.58 to −5.23)	−8.6 (−8.7 to −8.3)
Length of stay, d	3.9	−0.17 (−0.18 to −0.15)	−4.4 (−4.6 to −3.9)
Inpatient only	4.3	−0.27 (−0.29 to −0.26)	−6.4 (−6.7 to −6.1)
Share same- or next-day discharge, %	45.4	3.89 (3.70 to 4.07)	8.6 (8.2 to 9.0)
Share open approach, %^d^	43.0	−0.52 (−0.92 to −0.12)	−1.2 (−2.1 to −0.3)
Share discharged home, %	62.1	3.82 (3.65 to 3.99)	6.2 (5.9 to 6.4)
Inpatient only	51.2	3.41 (3.18 to 3.64)	6.7 (6.2 to 7.1)
Outpatient only	80.7	1.22 (0.99 to 1.44)	1.5 (1.2 to 1.8)
Postadmission			
30-d Mortality, per 1000 patients	2.9	0.15 (−0.07 to 0.37)	5.2 (−2.4 to 12.8)
Share readmitted, %^e^	11.6	−0.70 (−0.83 to −0.58)	−6.0 (−7.2 to −5.0)
30-d Estimated costs from admission, mean (SD), $^f^	21 942	−671 (−702 to −639)	−3.1 (−3.2 to −2.9)

Abbreviations: pp, percentage points; TM, traditional Medicare.

^a^ The TM mean is the contemporaneous, unadjusted mean of each surgical characteristic and outcome among TM surgical episodes. These values serve as the reference for interpreting the magnitude of the MA coefficients in the last column.

^b^ The coefficients come from estimating linear regressions that control for patients’ characteristics (age, sex, race, original reason for Medicare eligibility, dual Medicare-Medicaid eligibility, and Elixhauser Comorbidity Index for risk of in-hospital mortality), the surgery category, and the hospital referral region of the surgical facility.

^c^ Distance was measured between the centroids of the patient’s zip code and the surgical facility’s zip code.

^d^ Share of procedures that used an open approach, when a minimally invasive approach was also available in the surgery category.

^e^ Share of patients discharged alive from the surgical stay who had an emergency department visit or nonelective inpatient hospital admission within 30 days of discharge. The estimated regression for this outcome includes the Elixhauser Comorbidity Index for the risk of 30-day readmission rather than the index for risk of in-hospital mortality.

^f^ Costs were estimated using coefficients from a linear regression based on cost data for a random sample of 20% of TM patients. The regression estimating the correlation coefficient of MA enrollment included the Elixhauser Comorbidity Indexes for risk of 30-day readmission and for risk of in-hospital mortality.

During the surgical admission, MA patients had a shorter stay than comparable TM patients (difference, −0.17 [95% CI, −0.18 to −0.15] days), partly from a lower share of inpatient procedures (difference, −5.41 [95% CI, −5.58 to −5.23] pp) and partly because inpatient stays for MA patients were shorter than for TM patients. The percentage of patients discharged home was higher for those enrolled in MA vs TM (difference, 3.82 [95% CI, 3.65 to 3.99] pp), with both inpatient and outpatient procedures likely playing a role in lower use of postoperative care. Use of open vs minimally invasive alternatives was less common for MA patients (difference, −0.52 [95% CI, −0.92 to −0.12] pp).

Postoperatively, the proportion of MA patients with an emergency department or hospital readmission was lower (difference, −0.70 [95% CI, −0.83 to −0.58] pp). Mortality rates overall were very low for the surgical procedures included in our study, with no significant difference in 30-day mortality rates between patients with MA or TM.

Summing up the estimated 30-day costs, surgical episodes for MA patients were $671 (95% CI, $639-$702) lower compared with similar episodes for comparable TM patients. Costs were lower for patients with MA for all surgical categories (Figure and eTable 3 in Supplement 1). We found that 14% of the lower estimated cost in MA was attributed to between-facilities variation (eTable 2 in Supplement 1). After controlling for specific surgical codes instead of the surgical category, episodes for MA patients were less expensive ($360 [95% CI, $334-$386]) (eTable 4 in Supplement 1). MA cost savings were similar in a sensitivity analysis that controlled for patients’ HCC risk scores (eTable 5 in Supplement 1). Our findings remained unchanged when examining surgical procedures classified as urgent (eTable 6 and eTable 7 in Supplement 1), and when focusing only on patients with dual Medicare-Medicaid eligibility (eTable 8 in Supplement 1).

**Figure.**
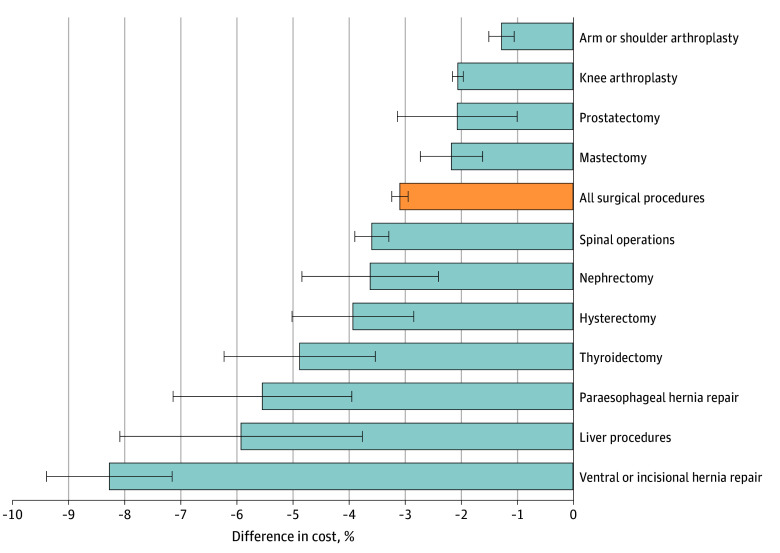
Differences in Estimated 30-Day Costs of Surgical Episodes for Medicare Advantage (MA) Patients Compared With Traditional Medicare (TM) Patients, by Surgical Category Difference in costs by surgical category for MA patients who underwent a surgical procedure and the costs of the procedure from the same category for similar TM patients (reference group). Correlation coefficients were calculated from linear regressions that estimated the correlation between MA enrollment of surgical patients in each surgical category and the estimated 30-day costs of the surgical episode. The model adjusted for patient characteristics (age, sex, race, original reason for Medicare eligibility, dual eligibility for Medicare and Medicaid, Elixhauser Comorbidity Index for risk of in-hospital mortality and Elixhauser Comorbidity Index for risk of 30-day readmission), and the hospital referral region. Error bars represent 95% CIs.

## Discussion

In this cohort study, we found that surgical episodes in MA patients had lower costs compared with similar surgical episodes for similar TM patients. A lower proportion of surgical procedures billed at higher inpatient rates and shorter inpatient hospital stays in MA patients played a role in the cost difference. Moreover, MA surgical patients were discharged home at a higher rate, avoiding additional postacute care spending, and they experienced lower readmission rates to hospitals or emergency departments. Taken together with the absence of a significant difference in 30-day mortality rates, these findings suggest that MA’s cost savings are not associated with lower quality of care.

Our findings suggest several mechanisms through which MA plans might achieve efficiencies for elective surgical procedures. First, our finding of a lower overall surgery rate for patients with MA is consistent with prior research.^4,5,13,14,15,16,17,18^ Overall lower surgery rates for MA patients could be associated with policies that MA plans adopt to reduce unnecessary use, including prior authorization or restricted availability of surgeons. However, lower surgical utilization rates could also represent an unmeasured selection of patients into MA. When examining rates by surgical categories, we found MA patients had higher rates of surgical procedures primarily performed to treat malignant neoplasms (including thyroidectomy, mastectomy, prostatectomy, and hysterectomy). These higher rates may reflect proactive preventive care and cancer screening in MA patients,^5,14^ potentially leading to increased detection and diagnosis rates of malignant neoplasms.

Second, our study contributes to the scant literature on resource utilization during surgical episodes. We found that the 30-day costs of surgical episodes in MA patients were 3.1% lower than similar episodes in TM patients. Surgical procedures in MA patients were more frequently performed in lower-cost settings, required shorter lengths of stay, had lower use of postacute care services, and had lower rates of readmissions. The reduction in the estimated surgical costs after controlling for specific surgical codes (eTable 4 in Supplement 1) instead of the surgical categories suggests that a substantial portion (46%) of cost savings with MA may result from directing patients toward lower-cost surgical procedures within each category, potentially through prior authorization requirements that favor outpatient surgical procedures. A simple calculation indicates that MA cost savings within surgical episodes amount to almost half of the savings from lower utilization of surgical procedures in MA. Our findings align with prior studies indicating a higher proportion of outpatient surgical procedures for MA patients^13^ and a lower use of postacute care after hospitalizations.^19^ Our findings partly contrast with those of another study that reported longer lengths of surgical stays for MA patients, although this study focused solely on inpatient admissions, including both elective and urgent surgical stays.^20^

Our estimates indicate that steering patients to specific facilities played a role in the observed results, accounting for 14% of the difference in estimated costs for surgical procedures in MA vs TM patients. Further evidence of patient steering arises from our finding that MA patients traveled longer distances to their surgical procedures compared with similar TM patients residing in the same zip code area, suggesting a role for selective contracting with surgical facilities and surgeons into MA networks. Selective contracting based on primary care physican costliness has been identified before in MA.^21^ The remaining within-facility differences between similar MA patients and TM patients may be attributed to the influence of MA plans’ care management programs as well as differences in payment and auditing practices.

The absence of a significant difference in 30-day mortality rates between MA patients and TM patients who underwent surgical procedures as well as the lower rates of readmissions for MA patients, suggests that cost savings were not achieved at the expense of quality. This finding aligns with prior studies that reported similar outcomes for TM and MA patients hospitalized for inpatient care.^15,17^

### Limitations

Our study had several limitations. First, despite our rich data and controls, it is possible that some unmeasured residual differences persisted between MA and TM patients. We found that surgical categories with lower utilization among MA patients compared with TM patients also tended to have smaller cost savings, which could reflect unobserved differences in patients’ medical complexity. If so, our estimates of lower episode costs for MA patients undergoing surgical procedures may be conservative.

Second, our data did not include information regarding MA payments. Consequently, we were unable to directly assess MA costs, and our cost estimates, in practice, projected what would have been the cost of each surgical episode had it been performed in TM patients based on the episode’s characteristics. These estimates relied on linear regressions, which may be sensitive to potential differences in the cost distributions and could not be validated due to the lack of actual MA cost data.

Third, our dataset included claims from stand-alone ASCs for a sample of only 20% of beneficiaries. While this random sample should be representative, our coverage of procedures in stand-alone ASCs was not complete. Nevertheless, the use of ASCs was limited or forbidden^22^ for the surgical procedures in our sample, limiting any potential bias.

Fourth, our study does not account for heterogeneity within TM (eg, participation in an Accountable Care Organization), or within MA (eg, variation by plan type or sponsor). Additionally, we were able to identify minimally invasive surgical alternatives in outpatient settings only when a separate *CPT* code existed. This limitation applied equally to surgical procedures for MA and TM patients.

## Conclusions

This study found that for common categories of elective surgical procedures, the costs of surgical episodes were lower for patients with MA vs TM. Compared with similar TM patients, MA patients had fewer inpatient surgical procedures, had shorter lengths of stay, and were more frequently discharged directly home without additional postacute care. The findings may be partly attributed to steering MA patients to outpatient facilities, but they also suggest an important role for MA plans’ care management programs. Lower perioperative costs and perioperative resource use highlight other potential mechanisms by which MA plans may achieve cost savings compared with TM.

## Notes

Supplement 1. **eTable 1.** Average 30-day costs after a surgical admission in the 20% TM sample, by surgery category **eTable 2**. Regression analysis – correlation of MA enrollment with surgery characteristics and outcomes, controlling for facility fixed effects **eTable 3. **Correlation of MA enrollment with surgery characteristics and outcomes, by surgery category **eTable 4.** Correlation of MA enrollment with surgery characteristics and outcomes, controlling for specific surgery codes instead of surgery categories **eTable 5**. Correlation of MA enrollment with surgery characteristics and outcomes, based on the 20% sample of beneficiaries, controlling for HCC risk scores **eTable 6.** Age- and gender-standardized surgery rates, by surgery category and Medicare program, inpatient-only urgent/emergent surgeries **eTable 7.** Regression analysis – correlation of MA enrollment with surgery characteristics and outcomes, inpatient-only urgent/emergent surgeries **eTable 8.** Regression analysis – correlation of MA enrollment with surgery characteristics and outcomes, dual-eligibles-only sample (112,683 procedures)

Supplement 2.
Data Sharing Statement
