# Supplementary material for: Perioperative Costs of Elective Surgical Procedures in Medicare Advantage Compared With Traditional Medicare
Source: JAMA Health Forum. 2025 Aug 1;6(8):e252258. doi: 10.1001/jamahealthforum.2025.2258 (PMC12317349; doi:10.1001/jamahealthforum.2025.2258)
Supplement: Supplement 1. — eTable 1. Average 30-day costs after a surgical admission in the 20% TM sample, by surgery category eTable 2. Regression analysis – correlation of MA enrollment with surgery characteristics and outcomes, controlling for facility fixed effects eTable 3. Correlation of MA enrollment with surgery characteristics and outcomes, by surgery category eTable 4. Correlation of MA enrollment with surgery characteristics and outcomes, controlling for specific surgery codes instead of surgery categories eTable 5. Correlation of MA enrollment with surgery characteristics and outcomes, based on the 20% sample of beneficiaries, controlling for HCC risk scores eTable 6. Age- and gender-standardized surgery rates, by surgery category and Medicare program, inpatient-only urgent/emergent surgeries eTable 7. Regression analysis – correlation of MA enrollment with surgery characteristics and outcomes, inpatient-only urgent/emergent surgeries eTable 8. Regression analysis – correlation of MA enrollment with surgery characteristics and outcomes, dual-eligibles-only sample (112,683 procedures) [file jamahealthforum-e252258-s001.pdf]

Supplemental Online Content

Politzer E, Anderson TS, Ayanian JZ, Curto VE, Souza J, Tsai TC, Landon BE. Perioperative Costs of Elective Surgeries in Medicare Advantage Compared With Traditional Medicare. *JAMA Health Forum.* 2025;6(8): e252258. doi: 10.1001/jamahealthforum.2025.2258

eTable 1. Average 30-day costs after a surgical admission in the 20% TM sample, by surgery category

eTable 2. Regression analysis – correlation of MA enrollment with surgery characteristics and outcomes, controlling for facility fixed effects

eTable 3. Correlation of MA enrollment with surgery characteristics and outcomes, by surgery category

eTable 4. Correlation of MA enrollment with surgery characteristics and outcomes, controlling for specific surgery codes instead of surgery categories

eTable 5. Correlation of MA enrollment with surgery characteristics and outcomes, based on the 20% sample of beneficiaries, controlling for HCC risk scores

eTable 6. Age- and gender-standardized surgery rates, by surgery category and Medicare program, inpatient-only urgent/emergent surgeries

eTable 7. Regression analysis – correlation of MA enrollment with surgery characteristics and outcomes, inpatient-only urgent/emergent surgeries

eTable 8. Regression analysis – correlation of MA enrollment with surgery characteristics and outcomes, dual-eligibles-only sample (112,683 procedures)

This supplemental material has been provided by the authors to give readers additional information about their work.

© 2025 Politzer E et al. *JAMA Health Network*.

eTable 1: Average 30-day costs after a surgical admission in the 20% TM sample, by surgery category

Surgery Group	Mean Total Costs, $	Of these: During the surgical stay, $	Mean Total Costs Breakdown				# of Obs.
			Inpatient Costs, %	Outpatient Costs, %	Professional Costs, %	Post-Acute Costs, %	
All Surgeries	21,035	15,635	59%	15%	17%	9%	161,567
Knee Arthroplasty	19,871	14,408	50%	21%	15%	14%	64,019
Spinal Surgeries	27,944	21,161	67%	9%	18%	7%	43,918
Arm/Shoulder Arthroplasty	22,121	18,101	76%	3%	13%	9%	12,922
Ventral/Incisional Hernia	12,282	7,212	47%	27%	21%	5%	12,060
Thyroidectomy	8,648	4,520	23%	45%	29%	2%	6,543
Prostatectomy	17,471	14,618	55%	23%	18%	4%	5,593
Mastectomy	13,086	8,562	17%	45%	31%	6%	4,467
Paraoesophageal Hernia Repair	17,187	14,244	38%	25%	17%	3%	3,624
Hysterectomy	18,730	15,241	66%	10%	18%	6%	3,176
Nephrectomy	21,898	17,890	72%	7%	15%	6%	3,417
Liver Procedures	30,629	23,444	74%	6%	16%	5%	1,828

© 2025 Politzer E et al. *JAMA Health Network*.

eTable 2: Regression analysis – correlation of MA enrollment with surgery characteristics and outcomes, controlling for facility fixed effects

	Surgery Characteristics and Outcomes of Interest	**Correlation Coeff. Of MA Enrollment** [a]	**95% CI**	**Share Attributed to Between-Facilities Variation, %** [b]
During Admission	**Share Inpatient, pp** [c]	**-3.66**	[-3.83,-3.49]	32%
	**Length of Stay, Days**	**-0.11**	[-.12,-.09]	37%
	**Inpatient only**	**-0.26**	[-.28,-.25]	3%
	**Same- or Next-Day Discharge, pp**	**2.43**	[2.25,2.61]	37%
	**Share Open Approach, pp** [d]	0.09	[-.32,.49]	117%
	**Discharge Home, pp**	**3.78**	[3.62,3.95]	1%
	**Inpatient only**	**3.46**	[3.23,3.68]	-1%
	**Outpatient only**	**1.79**	[1.59,2.00]	-48%
Post-Admission	**30-Days Mortality, per 1,000**	0.08	[-.14,.31]	45%
	**Share Readmitted, pp** [e]	**-0.81**	[-.94,-.68]	-15%
	**30-Days Predicted Costs, $** [f]	**-575**	[-607,-543]	14%

[a] The coefficient comes from estimating linear regressions that control for patients' characteristics (age, gender, race, original reason for Medicare eligibility, dual eligibility for Medicare and Medicaid, and Elixhauser index for risk of in-hospital mortality), the surgery category, and the hospital referral region (HRR) of the surgical facility. It also includes fixed effects for each surgical facility, identified by their National Provider Identifier (NPI). Surgical facilities include inpatient and outpatient hospital facilities, and stand-alone ASCs. With these fixed effects, the coefficients of MA enrollment should be interpreted as correlation of MA enrollment with the outcomes of interest, compared to similar TM patients undergoing surgery in the *same* facility.

[b] The share of correlation attributed to between-facility variation equals 1 minus the ratio of the coefficient from the specification that includes facility FE and the baseline specification (Table 3 in the main text). This ratio can be negative if the MA between-facilities correlation has an opposite sign relative to the coefficient from the baseline specification.

[c] Share of procedures billed as inpatient surgery.

[d] Share of procedures using an open approach, where minimally invasive approach is also available in the surgery category.

[e] Share of patients discharged alive from the surgical stay that have an ED visit or non-elective inpatient hospital admission within 30 days of discharge. The estimated regression for this outcome includes the Elixhauser index for risk of 30-day readmission, rather than the index for risk of in-hospital mortality.

[f] Predicted costs over the 30-day period from the beginning of the surgical episode. Costs are predicted using coefficients from a linear regression, based on cost data for a random sample of 20% of TM patients. The regression estimating the correlation coefficient of MA enrollment includes the Elixhauser index for risk of 30-day readmission, in addition to the index for risk of in-hospital mortality.

© 2025 Politzer E et al. *JAMA Health Network*.

eTable 3: Correlation of MA enrollment with surgery characteristics and outcomes, by surgery category

	Pre-Admission	During Admission					Post-Admission		
	**Distance to Facility, miles**	**Share Inpatient, pp**	**Length of Stay, Days**	**Same- or Next-Day Discharge, pp**	**Share Open Approach, pp**	**Share Discharged Home, pp**	**30-days mortality, per 1,000**	**Share Readmitted, pp**	**30-Days Predicted Costs, $**
**Knee Arthroplasty**	**3.64**	**-8.32**	**-0.20**	**5.39**	NA	**2.91**	0.15	**-0.67**	**-409.59**
[95% CI]	[2.59,4.69]	[-8.59,-8.04]	[-.23,-.18]	[5.11,5.68]	NA	[2.63,3.18]	[-0.08,0.37]	[-.86,-.48]	[-429,-390]
MA relative to TM baseline (%)	8.5%	-12.2%	-5.2%	13.2%	NA	6.4%	12.0%	-6.4%	-2.0%
TM Baseline	43.0	68.1	3.9	41.0	100.0	45.3	1.2	10.4	20,147
# of observations					483,715				
**Spinal Surgery**	**3.31**	**-3.38**	0.00	**1.61**	NA	**5.99**	0.19	**-0.59**	**-1,097.81**
[95% CI]	[1.85,4.78]	[-3.76,-3.00]	[-.03,.03]	[1.22,2.00]	NA	[5.63,6.36]	[-.27,.64]	[-.87,-.32]	[-1191,-1005]
MA relative to TM baseline (%)	6.1%	-5.3%	0.1%	4.1%	NA	9.3%	6.1%	-4.6%	-3.6%
TM Baseline	54.5	63.4	4.1	39.2	100.0	64.5	3.0	13.0	30,919
# of observations					286,464				
**Arm/Shoulder Arthroplasty**	**3.77**	**-5.56**	**-0.28**	**5.34**	NA	**1.79**	0.07	-0.09	**-279.80**
[95% CI]	[1.12,6.42]	[-5.84,-5.27]	[-.32,-.24]	[4.70,5.98]	NA	[1.21,2.37]	[-.57,.72]	[-.49,.31]	[-329,-230]
MA relative to TM baseline (%)	7.6%	-5.7%	-9.1%	8.7%	NA	2.5%	3.5%	-1.0%	-1.3%
TM Baseline	49.3	97.2	3.1	61.7	100.0	70.2	2.2	9.1	22,173
# of observations					97,389				
**Ventral/Incisional Hernia Repair**	**4.52**	**-4.97**	**-0.28**	**2.95**	**-1.32**	**5.72**	0.08	**-1.10**	**-1,103.43**
[95% CI]	[1.67,7.36]	[-5.63,-4.31]	[.34,-.21]	[2.25,3.66]	[-2.01,-.63]	[5.21,6.23]	[-1.05,1.20]	[-1.58,-.62]	[-1253,-954]
MA relative to TM baseline (%)	10.7%	-15.9%	-7.5%	5.1%	-2.0%	6.9%	1.4%	-8.9%	-8.2%
TM Baseline	42.2	31.2	3.7	57.9	66.3	83.5	5.8	12.3	13,482
# of observations					82,931				
**Thyroidectomy**	-3.85	**-1.96**	0.01	0.00	NA	**3.29**	-0.25	**-0.80**	**-439.41**
[95% CI]	[-8.73,1.03]	[-2.52,-1.40]	[-.06,.07]	[-.71,.70]	NA	[2.85,3.74]	[-1.04,0.53]	[-1.36,-.25]	[-561,-318]
MA relative to TM baseline (%)	-5.9%	-19.1%	0.3%	0.0%	NA	3.5%	-16.1%	-9.2%	-4.8%
TM Baseline	64.8	10.3	2.6	82.3	100.0	93.0	1.6	8.7	9,108
# of observations					46,050				
**Prostatectomy**	**-6.27**	**7.14**	**-0.26**	**3.85**	0.40	**1.48**	**1.92**	**-1.56**	**-358.73**
[95% CI]	[-11.30,-1.23]	[6.22,8.07]	[-.34,-.17]	[2.88,4.82]	[-.30,1.11]	[0.79,2.18]	[.30,3.53]	[-2.34,-.78]	[-545,-173]
MA relative to TM baseline (%)	-9.4%	11.9%	-5.9%	8.5%	2.8%	1.8%	34.1%	-8.7%	-2.0%
TM Baseline	67.0	60.2	4.4	45.5	14.5	84.3	5.6	18.0	17,553
# of observations					43,245				

© 2025 Politzer E et al. *JAMA Health Network*.

	Pre-Admission	During Admission					Post-Admission		
	Distance to Facility, miles	Share Inpatient, pp	Length of Stay, Days	Same- or Next-Day Discharge, pp	Share Open Approach, pp	Share Discharged Home, pp	30-days mortality, per 1,000	Share Readmitted, pp	30-Days Predicted Costs, $
**Mastectomy**	**7.10**	**-1.87**	**-0.08**	**2.27**	NA	**4.38**	-0.02	**-0.91**	**-284.99**
[95% CI]	[2.19,12.02]	[-2.66,-1.08]	[-.14,-.02]	[1.33,3.22]	NA	[3.61,5.15]	[-.90,.86]	[-1.56,-2.53]	[-358,-212]
MA relative to TM baseline (%)	16.6%	-11.2%	-2.8%	3.2%	NA	5.3%	-1.1%	-9.3%	-2.1%
TM Baseline	42.7	16.7	2.9	70.6	100.0	82.3	1.6	9.8	13,284
# of observations					36,152				
**Paraoesophageal Hernia Repair**	4.57	**-5.30**	**-0.11**	**1.42**	**1.67**	**1.20**	1.22	-0.82	**-920.94**
[95% CI]	[-1.01,10.14]	[-6.51,-4.09]	[-.21,-.01]	[.19,2.65]	[.71,2.63]	[.51,1.89]	[-.84,.33]	[-1.69,.05]	[-1186,-656]
MA relative to TM baseline (%)	9.0%	-10.7%	-2.8%	3.0%	9.4%	1.3%	19.8%	-6.1%	-5.5%
TM Baseline	51.0	49.6	3.9	47.8	17.8	91.5	6.2	13.5	16,800
# of observations					28,147				
**Hysterectomy**	0.73	**-2.46**	**-0.24**	**4.04**	-0.63	**3.18**	0.92	**-1.28**	**-730.88**
[95% CI]	[-5.08,6.53]	[-3.57,-1.35]	[-.35,-.14]	[2.84,5.25]	[-1.88,.63]	[2.22,4.15]	[-1.08,2.92]	[-2.16,-0.40]	[-933,-529]
MA relative to TM baseline (%)	1.4%	-3.2%	-5.1%	12.4%	-1.3%	3.9%	16.9%	-9.7%	-3.9%
TM Baseline	52.4	76.0	4.7	32.7	47.4	81.2	5.4	13.2	18,812
# of observations					25,714				
**Nephrectomy**	**-8.49**	0.20	**-0.26**	**2.12**	-0.09	**1.99**	-1.19	**-1.52**	**-779.19**
[95% CI]	[-14.45,-2.53]	[-.63,1.04]	[-.37,-.15]	[1.10,3.14]	[-1.31,1.12]	[1.00,2.97]	[-3.76,1.38]	[-2.47,-.57]	[-1042,-517]
MA relative to TM baseline (%)	-14.0%	0.2%	-5.0%	11.0%	-0.3%	2.5%	-11.8%	-9.5%	-3.6%
TM Baseline	60.8	88.0	5.2	19.3	34.3	80.8	10.0	16.1	21,764
# of observations					26,491				
**Liver Procedures**	-7.24	**-4.50**	**-0.65**	**2.95**	**-2.45**	**4.06**	-0.87	-0.03	**-1,768.24**
[95% CI]	[-17.03,2.55]	[-6.05,-2.95]	[-.88,-.41]	[1.37,4.53]	[-4.23,-.66]	[2.45,5.68]	[-6.42,4.67]	[-1.47,1.42]	[-2415,-1122]
MA relative to TM baseline (%)	-10.8%	-5.9%	-9.5%	11.6%	-4.5%	5.9%	-3.5%	-0.1%	-5.9%
TM Baseline	67.1	76.2	6.8	25.5	54.6	69.1	24.9	19.7	30,184
# of observations					13,800				

[a] The coefficients come from estimating, separately for each surgery category, linear regressions that control for patients' characteristics (age, gender, race, original reason for Medicare eligibility, dual eligibility for Medicare and Medicaid, and Elixhauser index for risk of in-hospital mortality), and the hospital referral region (HRR) of the surgical facility.
[b] Distance is measured between the centroids of the patient's ZIP code and the surgical facility's ZIP code.
[c] Share of procedures billed as inpatient surgery.
[d] Share of procedures using an open approach, where minimally invasive approach is also available in the surgery category.
[e] Share of patients discharged alive from the surgical stay that have an ED visit or non-elective inpatient hospital admission within 30 days of discharge. The estimated regression for this outcome includes the Elixhauser index for risk of 30-day readmission, rather than the index for risk of in-hospital mortality.
[f] Predicted costs over the 30-day period from the beginning of the surgical episode. Costs are predicted using coefficients from a linear regression, based on cost data for a random sample of 20% of TM patients. The regression estimating the correlation coefficient of MA enrollment includes the Elixhauser index for risk of 30-day readmission, in addition to the index for risk of in-hospital mortality.

© 2025 Politzer E et al. *JAMA Health Network*.

eTable 4: Correlation of MA enrollment with surgery characteristics and outcomes, controlling for specific surgery codes instead of surgery categories

	MA vs. TM Differences for Comparable Surgical Patients	**TM Mean (Baseline)**	**Correlation Coeff. Of MA Enrollment**	**95% Confidence Interval**	**MA relative to Baseline, %**
Pre-Admission	**Distance to Facility, Miles**	49.1	**1.91**	[1.02,2.79]	3.9%
During Admission	**Length of Stay, Days**	3.9	**-0.27**	[-.29,-.25]	-6.9%
	**Same- or Next-Day Discharge, pp**	45.4	**5.62**	[5.41,5.83]	12.4%
	**Discharge Home, pp**	62.1	**3.28**	[3.06,3.50]	3.3%
Post-Admission	**30-Days Mortality, per 1,000**	2.9	0.32	[-.00,.65]	11.0%
	**Share Readmitted, pp**	11.6	**-0.66**	[-.83,-.49]	-5.7%
	**30-Days Predicted Costs, $**	21,942	**-360**	[-386,-334]	-1.6%

© 2025 Politzer E et al. *JAMA Health Network*.

eTable 5: Correlation of MA enrollment with surgery characteristics and outcomes, based on the 20% sample of beneficiaries, controlling for HCC risk scores

	Surgery Characteristics and Outcomes of Interest	**TM Mean (Baseline)**	**Correlation Coeff. of MA Enrollment [a]**	**95% CI**	**MA relative to Baseline, %**
Pre-Admission	**Distance to Facility, Miles [b]**	49.3	**2.11**	[.56,3.67]	4.3%
During Admission	**Share Inpatient, pp [c]**	59.2	**-3.24**	[-3.63,-2.86]	-5.5%
	**Length of Stay, Days**	3.7	-0.03	[-.06,.01]	-0.7%
	**Inpatient Only**	4.3	**-0.26**	[-.30,-.22]	-6.2%
	**Same- or Next-Day Discharge, pp**	49.2	**1.30**	[.89,1.71]	2.6%
	**Share Open Approach, pp [d]**	45.0	-0.77	[-1.65,.11]	-1.7%
	**Discharge Home, pp**	58.0	**6.55**	[6.16,6.94]	11.3%
	**Inpatient Only**	51.3	**3.69**	[3.18,3.64]	7.2%
	**Outpatient Only**	67.8	**8.59**	[8.02,9.15]	12.7%
Post-Admission	**30-Days Mortality, per 1,000**	2.8	0.15	[-.31,.62]	5.5%
	**Share Readmitted, pp [e]**	11.5	**-0.61**	[-.89,-.34]	-5.3%
	**30-Days Predicted Costs, $ [f]**	21,993	**-679**	[-746,-611]	-3.1%

[a] The coefficients come from estimating linear regressions that control for patients' characteristics (age, gender, race, original reason for Medicare eligibility, dual eligibility for Medicare and Medicaid, and Elixhauser index for risk of in-hospital mortality), the surgery category, and the hospital referral region (HRR) of the surgical facility. Regressions also control for patients' HCC risk score (based on 2018 diagnoses). All regressions are estimated on the 20% random sample of Medicare beneficiaries.

[b] Distance is measured between the centroids of the patient's ZIP code and the surgical facility's ZIP code.

[c] Share of procedures billed as inpatient surgery.

[d] Share of procedures using an open approach, where minimally invasive approach is available in the surgery category.

[e] Share of patients discharged alive from the surgical stay that have an ED visit or non-elective inpatient hospital admission within 30 days of discharge. The estimated regression for this outcome includes the Elixhauser index for risk of 30-day readmission, rather than the index for risk of in-hospital mortality.

[f] Predicted costs over the 30-day period from the beginning of the surgical episode. Costs are predicted using coefficients from a linear regression, based on cost data for a random sample of 20% of TM patients. The regression estimating the correlation coefficient of MA enrollment includes the Elixhauser index for risk of 30-day readmission, in addition to the index for risk of in-hospital mortality.

© 2025 Politzer E et al. *JAMA Health Network*.

*eTable 6: Age- and gender-standardized surgery rates, by surgery category and Medicare program, inpatient-only **urgent/emergent** surgeries*

**Surgery Category**	**Total # of Procedures** [a]	**MA Rate, per 1,000** [b]	**TM Rate, per 1,000** [b]	**MA vs. TM Diff.**	**Diff. 95% CI**
All Sample	114,758	2.21	2.03	**0.18**	[.16,.21]
Knee Arthroplasty	15,198	0.31	0.25	**0.07**	[.06,.07]
Spinal Surgeries	35,532	0.66	0.63	0.03	[-0.0,.06]
Arm/Shoulder Arthroplasty	6,986	0.14	0.12	**0.01**	[.01,.02]
Ventral/Incisional Hernia	28,996	0.53	0.52	**0.01**	[-0.0,.02]
Prostatectomy	11,083	0.22	0.19	**0.03**	[.02,.04]
Paraoesophageal Hernia	3,941	0.07	0.07	0.00	[0.0,0.0]
Hysterectomy	3,220	0.07	0.05	0.01	[0.0,.02]
Nephrectomy	2,152	0.04	0.04	0.00	[0.0,0.0]
Liver Procedures	6,369	0.12	0.11	0.01	[0.0,.02]

*eTable 7: Regression analysis – correlation of MA enrollment with surgery characteristics and outcomes, inpatient-only **urgent/emergent** surgeries*

	Surgery Characteristics and Outcomes of Interest	**TM Mean (Baseline)**	**Correlation Coeff. of MA Enrollment** [a]	**95% CI**	**MA relative to Baseline, %**
Pre-Admission	**Distance to Facility, Miles** [b]	53.7	**6.03**	[1.62,3.01]	11.2%
During Admission	**Length of Stay, Days**	9.1	**-0.19**	[-.29,-.09]	-2.1%
	**Same- or Next-Day Discharge, pp**	7.6	**2.03**	[1.65,2.42]	26.7%
	**Share Open Approach, pp** [d]	55.8	-0.27	[-1.07,.54]	-0.5%
	**Discharge Home, pp**	37.9	**5.95**	[5.29,6.61]	15.7%
Post-Admission	**30-Days Mortality, per 1,000**	44.4	**-3.79**	[-6.67,-.92]	-8.5%
	**Share Readmitted, pp** [e]	21.1	-0.08	[-.67,.51]	-0.4%
	**30-Days Predicted Costs, $** [f]	43,561	**-766**	[-702,-639]	-1.8%

© 2025 Politzer E et al. *JAMA Health Network*.

eTable 8: Regression analysis – correlation of MA enrollment with surgery characteristics and outcomes, dual-eligibles-only sample (112,683 procedures)

	Surgery Characteristics and Outcomes of Interest	**TM Mean (Baseline)**	**Correlation Coeff. of MA Enrollment [a]**	**95% CI**	**MA relative to Baseline, %**
Pre-Admission	**Distance to Facility, Miles [b]**	35.2	**7.81**	[5.90,9.72]	22.2%
During Admission	**Share Inpatient, pp [c]**	66.9	**-5.71**	[-6.24,-5.17]	-8.5%
	**Length of Stay, Days**	4.4	**-0.15**	[-.20,-.10]	-3.4%
	**Inpatient Only**	5.0	**-0.24**	[-.30,-.18]	-4.8%
	**Same- or Next-Day Discharge, pp**	35.3	**3.90**	[3.33,4.47]	11.1%
	**Share Open Approach, pp [d]**	48.3	**-1.08**	[-.92,-.12]	-2.2%
	**Discharge Home, pp**	52.2	**6.28**	[5.72,6.83]	12.0%
	**Inpatient Only**	38.4	**6.72**	[6.02,7.42]	17.5%
	**Outpatient Only**	80.0	**1.14**	[.91,1.36]	1.4%
Post-Admission	**30-Days Mortality, per 1,000**	4.7	**-0.99**	[-.07,.37]	-2.1%
	**Share Readmitted, pp [e]**	17.0	0.37	[-.10,.83]	2.2%
	**30-Days Predicted Costs, $ [f]**	25,146	**-719**	[-702,-639]	-2.9%

© 2025 Politzer E et al. *JAMA Health Network*.
