# Supplementary material for: Perioperative Costs of Elective Surgical Procedures in Medicare Advantage Compared With Traditional Medicare
Source: JAMA Health Forum. 2025 Aug 1;6(8):e252258. doi: 10.1001/jamahealthforum.2025.2258 (PMC12317349; doi:10.1001/jamahealthforum.2025.2258)
Supplement: Supplement 2. — Data Sharing Statement [file jamahealthforum-e252258-s002.pdf]

Data Sharing Statement

Politzer. Perioperative Costs of Elective Surgical Procedures in Medicare Advantage Compared With Traditional Medicare. *JAMA Health Forum*. Published August 01, 2025. doi:10.1001/jamahealthforum.2025.2258

Data
Data available: No

Additional Information
Explanation for why data not available: Confidential CMS data.
